# Adolescent: provider connectedness and STI risk reduction following a brief alcohol intervention: findings from a randomized controlled trial

**DOI:** 10.3389/fpsyg.2023.1171264

**Published:** 2023-07-20

**Authors:** Genevieve F. Dash, Angela D. Bryan, Manshu Yang, Tammy Chung, Karen A. Hudson, Sarah W. Feldstein Ewing

**Affiliations:** ^1^Department of Psychological Sciences, University of Missouri, Columbia, MO, United States; ^2^Department of Psychology and Neuroscience, University of Colorado Boulder, Boulder, CO, United States; ^3^Department of Psychology, University of Rhode Island, Kingston, RI, United States; ^4^Department of Psychiatry, Institute for Health, Healthcare Policy and Aging Research, Rutgers, The State University of New Jersey, New Brunswick, NJ, United States

**Keywords:** adolescents, alcohol use, STI risk, common factors, motivational interviewing, mindfulness

## Abstract

**Objective:**

Given the frequent co-occurrence between alcohol use and sexual behavior among adolescents, alcohol interventions may play a role in helping prevent sexually transmitted infections (STIs) in this age group. Psychotherapy “common factors” are one potential active ingredient in intervention efficacy. Thus, the purpose of this study was to evaluate the influence of a critical common factor, adolescent: provider connectedness, on STI risk reduction at 3 months post-intervention.

**Methods:**

Community-based youth (*N* = 168) were randomized to two 60-min individual sessions of either motivational interviewing (MI) or brief adolescent mindfulness (BAM). Logistic regressions predicted post-intervention positive STI from adolescent: provider connectedness, intervention condition, and their interaction. Path analytic models tested post-intervention hazardous drinking as a mediator of the association between adolescent: provider connectedness and reduction in STI risk at 3-month follow-up.

**Results:**

Stronger adolescent: provider connectedness reduced risk of STI at 3 months post-intervention, with no differences by treatment condition. A mediational relationship between adolescent: provider connectedness and STI risk via hazardous drinking was not observed.

**Conclusion:**

Psychotherapeutic common factors, including adolescent: provider connectedness, may be important in mitigating adolescent health risk in behavioral interventions, above and beyond intervention condition and beyond the target behavior of the intervention.

## 1. Introduction

Adolescents represent one of the highest-risk groups for acquisition of sexually transmitted infections (STIs), accounting for nearly half of newly reported cases annually (Kreisel et al., 2021), and data continue to reflect that the likelihood of HIV infection is elevated among certain groups who have historically been less well represented in research, including youth ages 13–24 (Mendenhall and Singer, 2020). Of critical current public health importance is that present rates of oftentimes preventable STIs are escalating quickly among young people, despite several decades of declining rates of STIs in this age group (Feldstein Ewing and Bryan, 2020). Motivational interviewing (MI) has gained traction as an HIV/STI prevention intervention approach that is well-positioned to access and engage otherwise difficult-to-reach youth through settings such as pediatric/medical, juvenile justice, and school based-health centers (Vallabhan et al., 2017; D’Amico et al., 2018; Feldstein Ewing et al., 2022; Thompson et al., 2020; DiGuiseppi et al., 2021; Gaume et al., 2021; Miller et al., 2021; Sanchez-Puertas et al., 2022). Across settings, brief (often 1–2 session) HIV/STI prevention intervention programs such as MI have gained support for their capacity to reach and engage youth, often by meeting them where they are both physically and socioemotionally. Studies utilizing MI as a prevention program for youth HIV/STI and other health risk behaviors have shown reductions in health risk behaviors as distally as 12 months post-intervention (Murphy et al., 2012; D’Amico et al., 2018; Naar et al., 2020; Miller et al., 2021). While these existing HIV/STI prevention intervention approaches show promise, they still have modest effect sizes (Schmiege et al., 2011; Feldstein Ewing et al., 2016c; Bryan et al., 2018; Bryan and Feldstein Ewing, 2018; Gillman et al., 2018; Feldstein Ewing and Bryan, 2020; Gibson et al., 2020; Bryan et al., 2021; Schmiege et al., 2021). Even among the strongest evidence-based behavioral HIV/STI prevention interventions, including MI (Hettema et al., 2005; Lundahl et al., 2013; Miller and Rollnick, 2013; Henderson et al., 2020), effect sizes for youth (Jensen et al., 2011; Cushing et al., 2014; Steele et al., 2020; Calomarde-Gomez et al., 2021) indicate that there is still substantial room for improvement (Feldstein Ewing et al., 2016a; Silvers et al., 2019). Similarly, meta-analyses of MI reflect that gains are modest and component studies are fraught with statistical and clinical heterogeneity (Morales et al., 2018; Henderson et al., 2020). Together, these data leave concerned providers at a loss for how to better articulate HIV/STI prevention programs to more impactfully catalyze and sustain behavior change with this important and underserved age group (Cushing et al., 2014; Feldstein Ewing et al., 2016a,c; Morales et al., 2018; Silvers et al., 2019; Feldstein Ewing and Bryan, 2020; Steele et al., 2020).

Behavioral interventions for adolescent alcohol use may offer one avenue to more efficaciously target and reduce substance-related HIV/STI risk behavior (Kahler et al., 2018; Schmiege et al., 2021; Starks et al., 2022) by accessing and intervening on hazardous drinking-a central risk factor in the STI health decision making context (Feldstein Ewing et al., 2016c). Adolescence is a period of increased experimentation with alcohol, which frequently corresponds with debut and exploration of sexual behavior in this age group (Feldstein Ewing et al., 2016c). Alcohol use prior to intercourse is not uncommon: of the more than 27% of sexually active high school students in the United States (US), over one-fifth reported alcohol and/or other substance use prior to their most recent intercourse (Szucs et al., 2020). This is concerning, as alcohol use prior to intercourse can escalate risk for HIV/AIDS risk behaviors among adolescents, including acquisition of STIs (e.g., via incorrect condom use, condomless sex, and/or intercourse with multiple partners) (Ritchwood et al., 2015). Ultimately, when youth are intoxicated, they are less able to successfully engage in the requisite planning for enactment of health protective behaviors, which in turn increases the risk for exposure to STIs, and, in addition to other long-term sequelae (e.g., infertility, neurological problems, blindness), heightens risk of HIV infection (Feldstein Ewing et al., 2016c; Bryan et al., 2018; Feldstein Ewing and Bryan, 2020; DiClemente et al., 2021). A recent randomized controlled trial (RCT) found that the inclusion of alcohol content in a single-session, 2-h group-based HIV/AIDS risk intervention for adolescents reduced risk of STI at follow-up compared to an intervention focused only on reducing HIV/AIDS risk behavior (Bryan et al., 2018). Another study by the same team, but with a different sample, observed that MI interventions incorporating alcohol content were more efficacious in reducing HIV/AIDS risk behavior than an educational condition that only contained sexual risk reduction content (Bryan et al., 2021). These findings suggest that MI interventions incorporating alcohol content may be particularly well-positioned to reduce STI risk among youth.

### 1.1. The role of common therapeutic factors in efficacy of interventions for adolescent health risk behaviors

The therapeutic relationship is the cornerstone of MI, wherein empathic understanding and acceptance are critical to the delivery of the intervention (Moyers, 2014). A large literature has examined how these relational psychotherapeutic “common factors,” including adolescent: provider connectedness, may enhance outcomes (e.g., reductions in drinking) across intervention modalities (Wampold, 2015; Magill et al., 2019, 2021). Within the therapeutic context, the connection between patient and provider is assumed to be healing in and of itself (Cuijpers et al., 2019). Given the literature underscoring the particularly impactful role of therapeutic common factors in MI studies with adults more broadly (Miller and Moyers, 2015), it may be the case that adolescent: provider connectedness could also help enhance health risk reduction in the adolescent age group. This is particularly relevant given the developmentally salient shift in adolescents’ interpersonal awareness and social landscape that can directly impact health risk behavior (Silvers et al., 2019), changes in relationship dynamics with adults and authority figures (e.g., healthcare providers), and drive for increased autonomy over health-related behaviors and choices. Youth’s perceptions of the relationship between patient and provider play an important role in health-related decisions: youth are more likely to engage in mental healthcare if they feel respected, taken seriously, listened to, and not judged by their provider (Radez et al., 2021). Further, there is evidence that the therapeutic relationship is influential in outcomes of adolescent therapy for internalizing, externalizing, and substance-related problems (Shirk and Karver, 2003; Faw et al., 2005; McCambridge et al., 2011; McLeod, 2011; Shirk et al., 2011).

Common factors, including elements of the therapeutic relationship, are often posited to be at least partially responsible for the frequently observed lack of between-condition differences in efficacy across therapeutic modalities (Messer and Wampold, 2002). Our team’s prior clinical HIV/STI prevention intervention studies with youth have found fewer between-condition outcomes than expected, despite carefully and successfully ensuring distinction between modalities *via* use of separate therapists across conditions, separate supervision throughout the course of the study, and validated fidelity metrics that supported our capacity to deliver distinct content and clinical approaches in these interventions (Feldstein Ewing et al., 2013, 2014, 2015, 2016b, 2022; Mackiewicz-Seghete et al., 2022; Dash et al., 2023). As such, our observation of minimal differences between intervention conditions likely does not reflect intervention contamination and/or therapist overlap; rather, we posit that these outcomes reflect the presence and salience of common relational factors such as youth: provider relationship factors, and their impact across all modalities of HIV/STI prevention intervention programming (Miller and Moyers, 2015). Consistent with the broader common factors literature, it may be the case that these interventions are efficacious with adolescents because they provide 2 h of individual attention with a caring and nonjudgmental adult; that is, they foster a positive connection between the adolescent and the provider. This foundation of a warm, supportive, therapeutic environment could, in part, be what helps positively position youth for health-oriented behavior change.

### 1.2. Present study

The present study represents a secondary analysis of data from an RCT examining brief MI and mindfulness interventions for adolescent alcohol use (ClinicalTrials.gov registry number NCT03367858) The purpose of this study was to evaluate the influence of a common factor, adolescent: provider connectedness, on STI risk reduction at 3 months post-intervention across two therapeutic modalities: MI and brief adolescent mindfulness (BAM). Additionally, given the interconnected findings that (1) common factors within brief interventions for adolescents are associated with reductions in alcohol use, (2) many adolescents consume alcohol prior to engaging in sexual behavior, and (3) incorporating alcohol content into HIV/STI prevention intervention is associated with greater reductions in health risk behavior, we also aimed to test whether reductions in hazardous drinking might mediate the association between adolescent: provider connectedness and STI risk reduction. We hypothesized that adolescent: provider connectedness would significantly reduce odds of positive STI at 3-month follow-up above and beyond intervention condition, and that this negative association between adolescent: provider connectedness and positive STIs at 3-month follow-up would be mediated by post-intervention reductions in hazardous drinking.

## 2. Materials and methods

### 2.1. Trial design

The goal of the parent RCT was to begin to pave the way for new translational (integrated brain: behavioral) studies in the field of adolescent addiction (Mackiewicz-Seghete et al., 2022). Building upon prior work, which had largely used single-treatment arm within-subjects designs (Feldstein Ewing et al., 2013, 2016b), adolescents were randomized to one of two empirically supported behavioral treatments for addiction, MI (Jensen et al., 2011) and BAM (Crane et al., 2017). Participants across both conditions discussed factors relevant to problem drinking and received two individual 60-min sessions of one-on-one treatment contact; no treatment as usual (TAU) condition was administered. The MI and BAM interventions were selected due to the preliminary translational (integrated brain: behavioral) literature available for each modality-a topic central to the parent study questions (Mackiewicz-Seghete et al., 2022).

**Figure 1:**
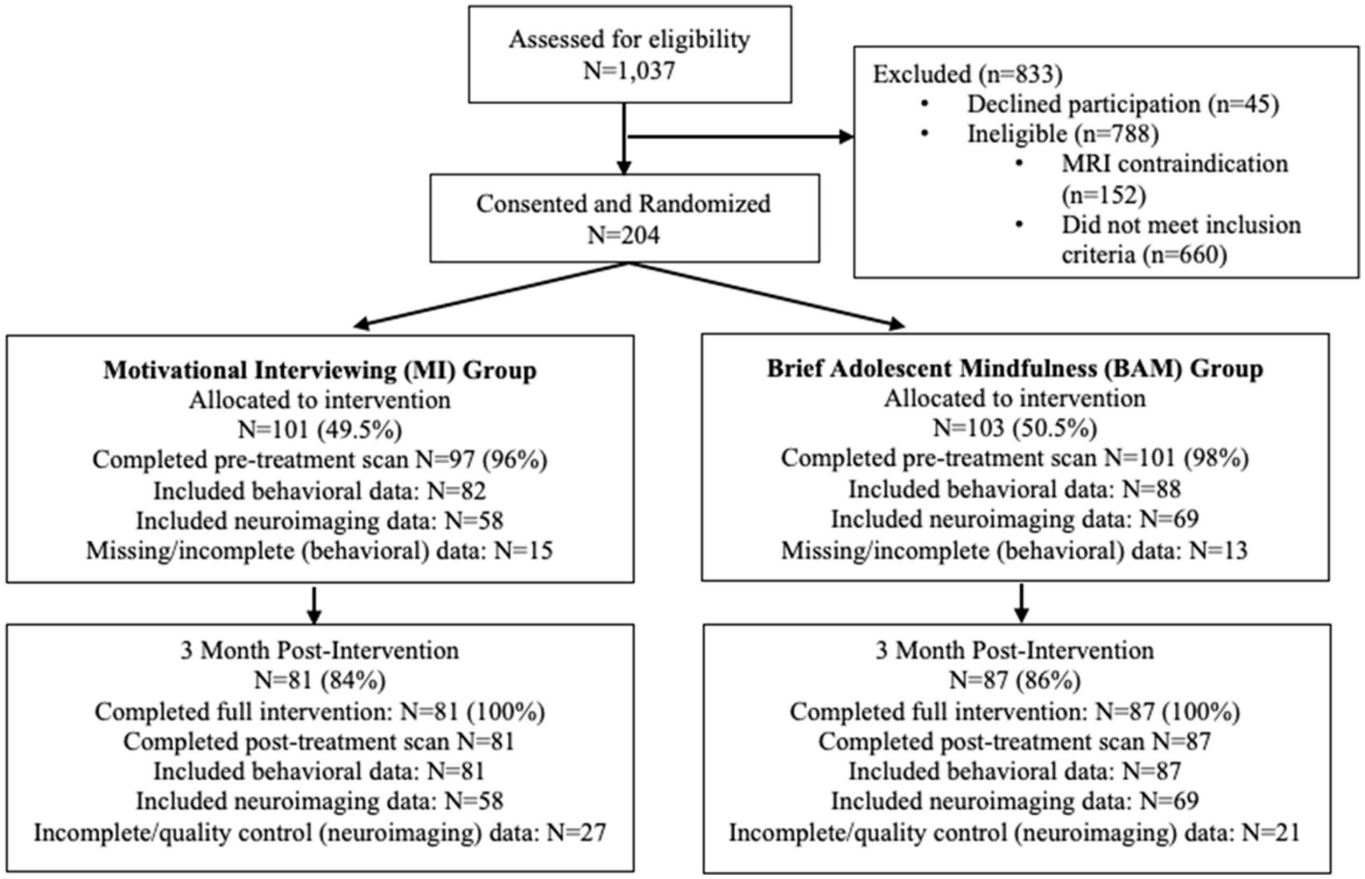
Study CONSORT.

The parent RCT utilized a parallel assignment model, with an allocation ratio of 1:1 (see CONSORT, Figure 1). Participants were blinded to intervention assignment and all interventionists were trained in, supervised in, and delivered only one of the two interventions. All study procedures were conducted with University Institutional Committee on Human Subjects approval and a federal Certificate of Confidentiality. Consent was obtained for participants age 18 years or older and parent consent with adolescent assent was obtained for youth under age 18 years. Youth received up to $150 for completing the intervention. Requests for deidentified data can be made to the senior author.

### 2.2. Participants

#### 2.2.1. Eligibility criteria

Participants were community-based youth recruited in the northwest United States. Eligibility included age of 14–19, current engagement in hazardous drinking (defined for the parent RCT as one or more heavy drinking episodes during past 2 months), and no more than three past-month non-tobacco or non-cannabis-substance use events. Exclusion criteria also included left-handedness and/or MRI contraindications in line with the translational requirements for the parent study.

#### 2.2.2. Present sample

Sample size for the parent study was determined *via* power analysis at a two-tailed alpha of 0.05 and power of.80. The power analysis assumed 16% attrition over the follow-up period. For this study, the analytic sample included all adolescents who completed the intervention and 3-month follow-up data collection protocol (*N* = 168; 35.71% female; M_age_ = 18.13 [SD = 1.12]; see Table 1). Baseline data were collected from participants at the university-based laboratory during attendance for intervention sessions, and follow-up was conducted in person at the university-based laboratory 3 months post-intervention.

**Table 1:** Sample characteristics and descriptive statistics.

	Total (*N* = 168)	MI (*N* = 81)	BAM (*N* = 87)	Difference test
Demographics	N (%) or M (SD)	N (%) or M (SD)	N (%) or M (SD)	
Age	18.13 (1.12)	18.35 (0.95)	17.92 (1.22)	***t* (166) = 2.40, *p* = 0.02**
Cisgender female	60 (35.71%)	30 (37.04%)	30 (34.48%)	χ^2^ (3) = 0.45, *p* = 0.93
Cisgender male	98 (58.33%)	47 (58.02%)	51 (58.62%)	
Transgender or genderqueer	10 (5.95%)	4 (4.94%)	6 (6.90%)	
Non-Hispanic white	129 (76.79%)	61 (75.31%)	68 (78.16%)	χ^2^ (6) = 5.30, *p* = 0.51
African American	8 (4.76%)	1 (1.23%)	7 (8.05%)	
Native Hawaiian or Pacific Islander	6 (3.57%)	2 (2.47%)	4 (4.60%)	
Asian American	28 (16.67%)	13 (16.05%)	15 (17.24%)	
Native American or Alaska Native	6 (3.57%)	2 (2.47%)	4 (4.60%)	
Hispanic	26 (15.48%)	17 (20.99%)	9 (10.34%)	
Other identity	5 (2.98%)	2 (2.47%)	3 (3.45%)	
Adolescent: provider connectedness	5.78 (1.22)	6.11 (1.04)	5.48 (1.30)	***t* (165) = −3.47 *p* = 0.0007**
Positive STI				
Positive STI history at baseline (lifetime)	11 (6.55%)	5 (7.81%)	6 (9.38%)	χ^2^ (1) = 0.10, *p* = 0.75
Positive STI at 3 month follow-up	6 (3.57%)	3 (4.48%)	3 (4.55%)	χ^2^ (1) = 0.53, *p* = 0.46
Hazardous drinking	4.45 (5.65)	4.93 (6.08)	4.01 (5.21)	*t* (166) = −1.05, *p* = 0.30

MI, motivational interviewing; BAM, brief adolescent mindfulness; STI, sexually transmitted infection; M, mean; SD, standard deviation. Bold font denotes statistically significant difference test.

### 2.3. Interventions

Adolescents were randomized to one of two individual-level manualized empirically supported behavioral treatments: MI (Feldstein Ewing et al., 2009) or BAM (Feldstein Ewing and Somohano, 2015). Of note, both MI and BAM are client-centered, primarily open-ended approaches (and thus, in these manuals, are youth-guided and not directive approaches). Participants received one of the two time-matched interventions. Interventions were comprised of two 60-min sessions that were delivered with at least one intervening weekend to give youth the opportunity to practice session content in between meetings. The MI intervention applied MI-congruent approaches (Miller and Rollnick, 2013) to foster adolescent-driven behavior change in the context of alcohol use. The adolescent-tailored mindfulness intervention introduced youth to eastern thought with the goal of demystifying mindful concepts to help unburden and navigate stressors, including in the context of alcohol use. Intervention manuals are available upon request to the senior author. Further details on the interventions, therapist training, and intervention fidelity are available in Mackiewicz-Seghete et al. (2022).

### 2.4. Outcome measures

Primary outcome measures for the parent RCT are described in Mackiewicz-Seghete et al. (2022). Results indicated that both interventions (MI and mindfulness) performed equivalently in reducing adolescent alcohol use at 3 months post-intervention, with no differences by treatment group. The design of the parent trial did not include collection of any information on harms of the intervention.

#### 2.4.1. Adolescent: provider connectedness

Immediately following the second and final intervention session, youth completed the Inclusion of Other in the Self scale, a pictorial measure of interpersonal closeness implemented in evaluations of behavioral interventions (Aron et al., 1992) that has been validated and used previously in adolescent populations (Lourenco et al., 2015; Braams and Crone, 2017; Meng et al., 2022). The measure includes a sequence of 7 images depicting two circles (one representing the youth and, for the present study, one representing the counselor) that overlap to varying degrees. Youth were instructed to select the image that they felt best represented their connectedness with their counselor. The scale ranged from 1 to 7. The adolescent: provider connectedness variable was mean centered for analysis.

#### 2.4.2. Sexually transmitted infection (STI)

At baseline, participants responded to a validated sexual history measure regarding lifetime history of STIs with the item: “have you ever had a sexually transmitted infection like chlamydia, herpes, or warts?” At the 3-month-follow-up, participants reported if they had been diagnosed with an STI (such as chlamydia, herpes, or warts) during the prior 3 months (i.e., since completing the intervention).

#### 2.4.3. Adolescent hazardous drinking

Adolescent hazardous drinking was measured by the Rutgers Alcohol Problems Index (RAPI; White and Labouvie, 1989). The RAPI is a well-validated 23-item metric of problem drinking (e.g., “Missed out on things because you spend too much money on alcohol”). Response options for each item (never, 1–2 times, 3–5 times, 6–10 times, more than 10 times) were summed to create an index of hazardous drinking. At follow-up, participants reported their post-intervention hazardous drinking over the past 3 months (*α* = 0.84) (i.e., since completing the intervention).

### 2.5. Statistical methods

Analyses were conducted in SAS Version 9.4 (SAS Institute Inc., 2014) and Mplus version 8 (Muthén, 2017). Preliminary analyses tested baseline equivalence on demographic characteristics, adolescent: provider connectedness, positive STI (lifetime), and hazardous drinking across conditions. Next, a series of logistic regression models predicting post-intervention STI risk reduction were fit using SAS PROC LOGISTIC. First, we tested a model including main effects of adolescent: provider connectedness and intervention, and an adolescent: provider connectedness x intervention interaction (“Model 1”). Next, the adolescent: provider connectedness x intervention interaction was dropped from the model (“Model 2”). Finally, positive STI (lifetime) at baseline was included as a predictor to determine whether the effect of adolescent: provider connectedness persisted above and beyond the effect of lifetime STI history (“Model 3”). We subsequently tested post-intervention hazardous drinking as a mediator of adolescent: provider connectedness and STI risk reduction at 3 months *via* path analysis conducted in Mplus. This model was structured to mirror Model 3, meaning that it included positive STI history (lifetime) at baseline and intervention as well as (1) a direct path from adolescent: provider connectedness to post-intervention STI risk reduction, (2) a direct path from adolescent: provider connectedness to post-intervention hazardous drinking, (3) a direct path from post-intervention hazardous drinking to post-intervention STI risk reduction, and (4) and indirect path from adolescent: provider connectedness to post-intervention STI risk reduction *via* post-intervention hazardous drinking. Modeling was conducted using full information maximum likelihood estimation with bootstrapped standard errors.

## 3. Results

### 3.1. Sample characteristics

Data collection was conducted from January 2017 through January 2020. All follow-up data were collected prior to the onset of the COVID-19 pandemic. Of the 1,037 youth screened for eligibility, 204 provided consent/assent and were randomized to condition (101 MI, 103 mindfulness). Of those participants, 168 completed 3-month follow-up data collection protocols (81 MI, 87 mindfulness; see Figure 1) and were included in the present analyses (results from analyses including all participants randomized to condition are available in the Supplemental material); one participant was excluded from analyses due to extreme response patterns. Demographics and descriptive statistics for study variables are presented in Table 1; SGM youth represented 6% of the sample. Baseline age and adolescent: provider connectedness differed across intervention groups, with participants in the MI condition being slightly older (18.35 years vs. 17.92 years) and reporting stronger adolescent: provider connectedness scores (6.11 vs. 5.48). Rates of positive STI did not differ across the MI and mindfulness conditions at baseline (7.81% vs. 9.38%) or 3-month follow-up (4.48% vs. 4.55%).

### 3.2. Model results

Results of the logistic regression models are presented in Table 2. In the first model (“Model 1”), stronger adolescent: provider connectedness was associated with lower odds of positive STI at 3 months post-intervention. The adolescent: provider connectedness x intervention interaction was nonsignificant, and inspection of the odds ratios for each condition confirmed that the effect of adolescent: provider connectedness on STI risk reduction at 3 months did not differ across intervention condition. As such, we proceeded with dropping the adolescent: provider connectedness x intervention interaction term from the model (“Model 2”), but retained intervention due to differences in adolescent: provider connectedness ratings across condition (see Table 1). The effect of adolescent: provider connectedness remained significant in this model, with higher adolescent: provider connectedness rating decreasing odds of positive STI at 3 months. When lifetime history of STI (baseline) was included in the model (“Model 3), adolescent: provider connectedness was, by a very small margin (0.04 vs. 07), no longer significant; however, magnitude of effect for adolescent: provider connectedness was not diminished, suggesting that this shift may be due increased imprecision of the estimate (as evidenced by the widened confidence interval), potentially resulting from data sparsity, rather than true absence of effect (Gelman and Stern, 2006; Greenland et al., 2016).

**Table 2:** Results from logistic regressions predicting positive STI at 3 months post-intervention.

	Estimate (95% CI)	OR (95% CI)	χ^2^	*p*
Model 1				
Main effect				
**Adolescent: provider connectedness**	**−0.58 (−1.10, −0.06)**	**-**	**4.69**	**0.03**
Intervention MI vs. BAM [Adolescent: provider connectedness = 0 (mean)]	−0.17 (−1.04, 0.70)	0.71 (0.13, 4.02)	0.15	0.70
Interaction				
Adolescent: provider connectedness x intervention	−0.13 (−0.65, 0.39)		0.25	0.62
MI		0.49 (0.21, 1.14)		
BAM		0.64 (0.35, 1.19)		
Model 2				
Main effect				
**Adolescent: provider connectedness**	**−0.54 (−1.04, −0.04)**	**0.58 (0.35, 0.97)**	**4.39**	**0.04**
Intervention	−0.07 (−0.85, 0.70)	0.86 (0.18, 4.09)	0.04	0.85
Model 3				
Main effect				
Adolescent: provider connectedness	−0.58 (−1.21, 0.05)	0.56 (0.30, 1.05)	3.24	0.07
Intervention	−0.53 (−1.61, 0.55)	0.35 (0.04, 3.02)	0.92	0.34
**No positive STI history at baseline (lifetime)**	**−1.92 (−2.94, −0.90)**	**0.02 (0.00, 0.16)**	**13.72**	**0.0002**

Bold font, significant estimate, *p* < 0.05; MI, motivational interviewing; BAM, brief adolescent mindfulness; STI, sexually transmitted infection; OR, odds ratio; CI, confidence interval.

**Figure 2:**
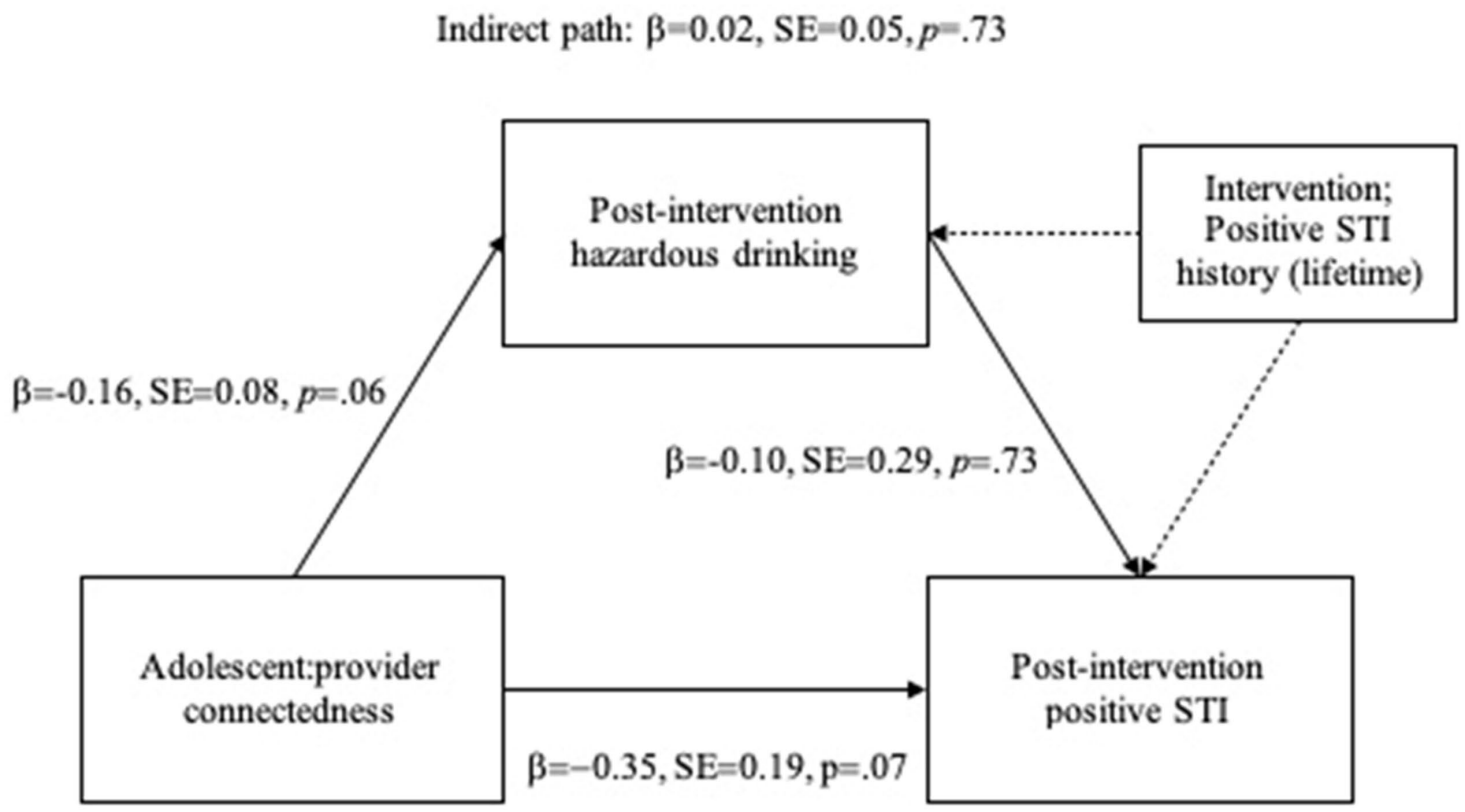
Mediation model.

Results from the mediation model are depicted in Figure 2. Post-intervention hazardous drinking did not predict STI risk reduction at 3 months (*β* = −0.10, SE = 0.29, *p* = 0.73). Direct effects of adolescent: provider connectedness on hazardous drinking neared but did not achieve statistical significance (*β* = −0.16, SE = 0.08, *p* = 0.06). The indirect effect adolescent: provider connectedness on STI risk reduction at 3 months via hazardous drinking was also near-zero and nonsignificant (*β* = 0.02, SE = 0.05, *p* = 0.73). Again, positive lifetime history of STI (baseline) was the most robust predictor of STI risk reduction at 3 months (*β* = 0.46, SE = 0.15, *p* = 0.002), though the direct effect of adolescent: provider connectedness displayed an estimate of moderate magnitude and neared statistical significance (*β* = −0.35, SE = 0.19, *p* = 0.07). Together, this pattern of results suggests that the present analysis may have been underpowered to detect the direct effects on adolescent: provider connectedness on post-intervention hazardous drinking and STI risk reduction within this more complex model, despite evidence of these associations in the prior models presented here.

## 4. Discussion

The present study aimed to explore the degree to which a well-established psychotherapeutic “common factor,” adolescent: provider connectedness, may impact adolescents’ HIV/AIDS risk behavior in the context of two widely-used brief behavioral interventions, MI and mindfulness. While most studies examining relational factors have done so in adult samples (Flückiger et al., 2018; Cuijpers et al., 2019), results here supported the role of common factors on HIV/AIDS outcomes during behavioral interventions for adolescents. Specifically, we found that adolescent: provider connectedness was significantly and directly associated with short-term (3 month) STI risk reduction among adolescents, an age group that is at elevated risk for STI and HIV/AIDS (Kreisel et al., 2021). In fact, adolescent: provider connectedness predicted STI risk reduction above and beyond the impact of each independent intervention (MI vs. mindfulness). This pattern of results indicates that adolescent: provider connectedness is relevant to health behavior outcomes beyond the sphere of substance use, even within an intervention targeting alcohol consumption. Given the role of positive STIs as a central risk factor for the later acquisition of HIV/AIDS, this finding has high public health relevance. Ultimately, these data suggest that providers who work with youth in caring, compassionate ways even during very brief (2-session) interventions, may be able to significantly impact the reduction of important health risk behavior at a time when STIs are on the rise among adolescents (Feldstein Ewing and Bryan, 2020).

In terms of clinical implications, the data observed in this study align with recent reviews (Feldstein Ewing et al., 2016a; Yeager et al., 2018; Silvers et al., 2019; Feldstein Ewing and Bryan, 2020), which suggest stepping back from existing paradigms to understand the nature and impact of the youth: provider relationship on youth HIV/STI and alcohol use outcomes in this age group. These clinical reviews support that interventions like MI allow providers to rapidly and impactfully connect with youth (Feldstein and Ginsburg, 2007). Qualitative data from our team’s prior studies reflect that youth report feeling like these brief prevention intervention programs offer an opportunity to explore their HIV/STI and related syndemic health risk behavior in a non-judgmental atmosphere, facilitating the unique experience of feeling listened to, respected, and empowered to make a behavioral change when they are ready. Similarly, providers conducting brief prevention programs with youth can clearly articulate those with whom they felt more connected, and how that connection seemed to act as an engine for therapeutic rapport and subsequent HIV/STI and related health risk intervention gains. While it is clear to most MI providers that these relational factors represent an essential component of who responds (and who does not) to brief behavioral HIV/STI prevention intervention programs (Miller and Moyers, 2015), this study takes a step further by examining these relationships quantitatively with understudied an underserved age group.

Such empirical data are urgently needed to meaningfully advance provider direct practice in HIV/STI and syndemic prevention intervention programs with youth (Feldstein Ewing et al., 2016a; Silvers et al., 2019; Feldstein Ewing and Bryan, 2020). We propose that we have found comparable outcomes across distinct evidence-based behavioral HIV/STI and syndemic prevention interventions (MI vs. mindfulness) within our RCTs because they all share the common core of providing youth with individualized attention with a caring adult. Important in this equation, these relationships were not localized to only HIV/STI and syndemic prevention interventions utilizing approaches specific to MI or mindfulness; rather, these effects have also been generalized across other intervention modalities examined by our team, including those with highly didactive/tutorial-based and reward-centered frameworks, such that they performed on par with the youth receiving MI at some, if not all, of the study follow-ups (Feldstein Ewing et al., 2013, 2014, 2015, 2016b, 2022; Mackiewicz-Seghete et al., 2022; Dash et al., 2023). In sum, there appears to be something highly impactful in this youth: provider relationship that we must continue to explore in youth clinical research.

This has several implications for risk reduction strategies for adolescents and training for providers who work with this age group. These data suggest that a relationship with a caring adult can reduce youth engagement in HIV/AIDS risk behaviors, even within an intervention focused on other health risk behavior (i.e., alcohol use). While future research is requisite to disaggregate what constitutes meaningful therapeutic rapport, and how we can best facilitate and achieve it with our youth who are of high need and low treatment receipt, this study indicates that opening the door to build an impactful relationship is critical. The potential transportability of such a transtheoretical approach is highly promising in terms of generalizing risk reduction approaches to a wide range of settings and maximizing the reach of efficacious approaches. In terms of training for providers, an important implication may be that explicit training in skills that foster interpersonal connectedness in addition to the “nuts and bolts” of manualized treatments is of critical importance.

### 4.1. Limitations

While this study had numerous strengths, including a first look at the role of the adolescent: provider connectedness in STI outcomes within an underexamined age group, results of the present study should be interpreted in light of limitations. Because the parent study was not originally developed to examine STI risk reduction, we were not able to include biometric testing for STIs. In addition, given the somewhat limited sample size, analyses may have been underpowered; future studies would benefit from replication of this analysis with a larger sample size fully powered to detect what is often a subtle effect for therapeutic outcomes this age group. Relatedly, it is unclear how results from this sample of adolescents recruited from the northwest US may generalize to other regions and populations. Finally, while it speaks well to our teams’ capacity to engender positive therapeutic working relationships with this sample of young people, ratings of adolescent: provider therapeutic connectedness were uniformly high, which may have limited variability to detect statistically significant associations between study variables.

### 4.2. Conclusion

This study builds on recent calls regarding the importance of explicitly examining the multifactorial nature of HIV risk in order to specifically incorporate and examine co-occurring outcomes that can dynamically exacerbate youth health risk, including the intersection of sexual risk behavior, positive STI, and alcohol use. The importance of examining such syndemic outcomes simultaneously is that they can interactively, negatively influence young people’s developmental trajectory, placing youth at greater risk for sustained patterns of health risk and related problems as they transition into adulthood (Castelpietra et al., 2022; Fu et al., 2022; Safiri et al., 2022). Extant meta-analyses of patient: provider relational factors indicate promise (small to medium effects) in broad-based mental health outcomes among older populations (e.g., adults) (Martin et al., 2000; Rouleau et al., 2020; Del Re et al., 2021; Rodriguez et al., 2021; Lauckner et al., 2022) and children/adolescents (Murphy and Hutton, 2018; Roest et al., 2023), but the youth HIV/STI prevention intervention field remains largely absent from empirical studies that examine the role of youth: provider relational factors. The present study is one step toward filling this gap.

Given that behavioral interventions are one of the most widely utilized intervention approaches to reduce HIV/AIDS risk for adolescents (Cushing et al., 2014; Hosek and Pettifor, 2019), these findings represent a relevant signal indicating the importance of further future exploration of the role of common factors across other adolescent HIV/AIDS risk reduction modalities. While these data do not ask or answer questions that enable us to speak to risk reduction strategies that may be most impactful to adolescents at this time, these data do highlight the continued need to deeply query and evaluate what constitutes common factors and how they may be impactful in HIV/STI risk reduction.

Because adolescence is a time of enhanced exploration of health risk behaviors including alcohol use and HIV/AIDS risk behaviors, identifying how to most meaningfully promote therapeutic relationships that will allow practitioners to explore HIV/AIDS health during what is often limited contact with clinical providers and youth is of critical public health importance for high need and underserved young people (Feldstein Ewing and Bryan, 2020). This study suggests that even in brief intervention settings, individual time with a caring adult may be particularly impactful in supporting health-oriented adolescent behavior change across numerous domains of adolescent health, including, but not limited to, HIV/AIDS risk reduction. Future research should continue to build upon these findings to examine how common factors within other types of brief and/or behavioral intervention modalities may continue to impact adolescent health and development. In sum, this study begins to open an essential window into the role of relational factors in youth HIV/STI prevention intervention response-an under-studied area among youth at high risk for HIV. Together, these data bring us one small step closer to developing more impactful HIV/STI prevention interventions, delivered at the right time, in the right way, to high-risk young people.

## Data availability statement

The data analyzed in this study is subject to the following licenses/restrictions: the data that support the findings of this study are available from the senior author upon reasonable request. Requests to access these datasets should be directed to SWFE, feldsteinewing@uri.edu.

## Ethics statement

This study involved human participants and was reviewed and approved by the Institutional Review Boards at Oregon Health & Science University and the University of Rhode Island. Written informed consent to participate in this study was obtained from participants (age 18 or older) or from the participants’ legal guardian with participant assent (under age 18).

## Author contributions

SWFE secured funding and collected the data used in the present study. SWFE and GFD contributed to the conceptualization of the study. GFD conducted data analysis. GFD drafted the first version of the manuscript. All authors contributed to editing the manuscript and revising it for important intellectual content. All authors provided approval for publication of the content of the manuscript and agree to be accountable for all aspects of the work in ensuring that questions related to the accuracy or integrity of any part of the work are appropriately investigated and resolved.

## Funding

This work was supported by the National Institute on Alcohol Abuse and Alcoholism [grant numbers 1R01AA023658–01, K24AA026876–0 (to SWFE)] and the National Institute on Drug Abuse [grant number F31DA054701 (to GFD)].

## Conflict of interest

The authors declare that the research was conducted in the absence of any commercial or financial relationships that could be construed as a potential conflict of interest.

## Publisher’s note

All claims expressed in this article are solely those of the authors and do not necessarily represent those of their affiliated organizations, or those of the publisher, the editors and the reviewers. Any product that may be evaluated in this article, or claim that may be made by its manufacturer, is not guaranteed or endorsed by the publisher.
