# Supplementary material for: Adolescent: provider connectedness and STI risk reduction following a brief alcohol intervention: findings from a randomized controlled trial
Source: Front Psychol. 2023 Jul 20;14:1171264. doi: 10.3389/fpsyg.2023.1171264 (PMC10399588; doi:10.3389/fpsyg.2023.1171264)
Supplement: Supplementary file 1 [file Table_1.DOCX]

**Supplemental Materials**

| Table S1. *Results from logistic regressions predicting positive STI at 3 months post-intervention within all participants randomized to condition (N=204)* | | | | |
| --- | --- | --- | --- | --- |
|  | Estimate (95% CI) | OR (95% CI) | χ^2^ | *p* |
|  | Model 1 | | | |
| Main effect |  |  |  |  |
| **Adolescent:provider connectedness** | **-0.40 (-0.78-, -0.02)** | **-** | **4.29** | **.04** |
| Intervention MI vs. BAM  (Adolescent:provider connectedness=0 [mean]) | -0.06 (-0.69, 0.57) | 0.89 (0.25, 3.13) | 0.03 | .86 |
| Interaction |  |  |  |  |
| Adolescent:provider connectedness x intervention | -0.02 (-0.40, 0.36) |  | 0.01 | .90 |
| MI |  | 0.69 (0.40, 1.18) |  |  |
| BAM |  | 0.66 (0.38, 1.12) |  |  |
|  | Model 2 | | | |
| Main effect |  |  |  |  |
| **Adolescent:provider connectedness** | **-0.40 (-0.78, -0.02)** | **0.67 (0.46, 0.98)** | **4.27** | **.04** |
| Intervention | -0.04 (-0.63, 0.54) | 0.92 (0.29, 2.95) | 0.02 | .89 |
|  | Model 3 | | | |
| Main effect |  |  |  |  |
| Adolescent:provider connectedness | -0.48 (-1.06, 0.11) | 0.62 (0.35, 1.12) | 2.50 | .11 |
| Intervention | -0.44 (-1.30, 0.43) | 0.42 (0.07, 2.36) | 0.98 | .32 |
| **No positive STI history** **at baseline (lifetime)** | **-2.33 (-3.25, -1.40)** | **0.01 (0.00, 0.06)** | **24.37** | **<.0001** |
| Note. Bold font=significant estimate, *p*<.05; MI=motivational interviewing; BAM=brief adolescent mindfulness; STI=sexually transmitted infection; OR=odds ratio; CI=confidence interval. | | | | |
